# Role of insula and its subregions in progression from recent onset to chronic idiopathic tinnitus

**DOI:** 10.1093/braincomms/fcad261

**Published:** 2023-10-07

**Authors:** Qian Chen, Han Lv, Zhaodi Wang, Xiaoshuai Li, Xinghao Wang, Yuyou Huang, Pengfei Zhao, Zhenghan Yang, Shusheng Gong, Zhenchang Wang

**Affiliations:** Department of Radiology, Beijing Friendship Hospital, Capital Medical University, Beijing 100050, China; Department of Radiology, Beijing Friendship Hospital, Capital Medical University, Beijing 100050, China; Department of Otolaryngology, Beijing Jingmei Group General Hospital, Beijing 102300, China; Department of Radiology, Beijing Friendship Hospital, Capital Medical University, Beijing 100050, China; Department of Radiology, Beijing Friendship Hospital, Capital Medical University, Beijing 100050, China; Qiyuan Lab, Beijing 100086, China; Department of Radiology, Beijing Friendship Hospital, Capital Medical University, Beijing 100050, China; Department of Radiology, Beijing Friendship Hospital, Capital Medical University, Beijing 100050, China; Department of Otolaryngology Head and Neck Surgery, Beijing Friendship Hospital, Capital Medical University, Beijing 100050, China; Department of Radiology, Beijing Friendship Hospital, Capital Medical University, Beijing 100050, China

**Keywords:** recent-onset tinnitus, chronic tinnitus, local neural activity, grey matter, functional connectivity

## Abstract

We determined the structural and functional alterations in the insula and its subregions in patients with idiopathic tinnitus in order to identify the neural changes involved in the progression from recent onset to chronic tinnitus. We recruited 24 recent-onset tinnitus patients, 32 chronic tinnitus patients and 36 healthy controls. We measured the grey matter volume and fractional amplitude of low-frequency fluctuation of the insula and its subregions and the functional connectivity within the insula and between the insula and the rest of the brain. Relationships between MRI and clinical characteristics were estimated using partial correlation analysis. Both recent-onset and chronic tinnitus patients showed decreased fractional amplitude of low-frequency fluctuation in the insula and its subregions, but only chronic tinnitus patients showed bilateral grey matter atrophy in the ventral anterior insula. Abnormal functional connectivity was detected in recent-onset and chronic tinnitus patients relative to the healthy controls, but functional connectivity differences between recent-onset and chronic tinnitus patients were found in only the auditory-related cortex, frontal cortex and limbic system. Functional alterations (fractional amplitude of low-frequency fluctuation and functional connectivity of the left ventral anterior insula), but not structural changes, were correlated with clinical severity. Bilateral grey matter atrophy in the ventral anterior insula decreased regional activities in the left ventral anterior insula and left posterior insula, and abnormal functional connectivity of the insula subregions with auditory and non-auditory areas were implicated in the progression from recent onset to chronic tinnitus. This suggests that tinnitus generation and development occur in a dynamic manner and involve aberrant multi-structural and functional (regional brain activity and abnormal functional connectivity) reorganization of the insula.

## Teaser

Chen *et al*. report that, by investigating structural and functional alterations in the insula and its subregions in patients with recent-onset and chronic tinnitus, they found that tinnitus generation and development occur dynamically and involve aberrant multi-structural and functional (regional brain activity and abnormal functional connectivity) reorganization of the insula.

## Graphical Abstract

**Graphical Abstract:**
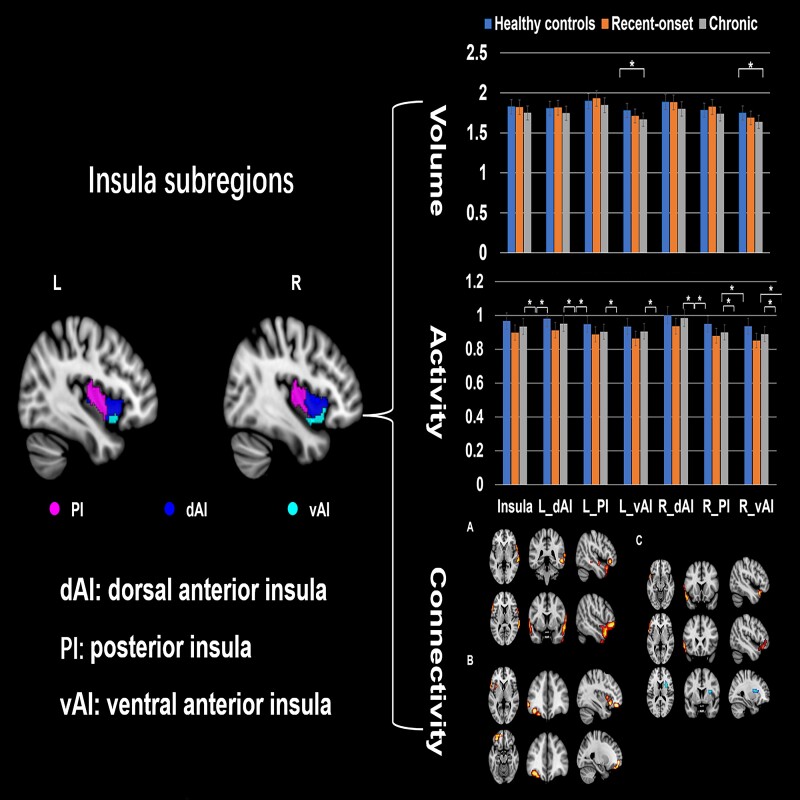

## Introduction

Tinnitus is a perception of sound without any internal or external stimulus. Tinnitus is a common condition, with an incidence of approximately 10–15% in adults.^1^ Tinnitus and its negative consequences, for example, anxiety and depression, greatly diminish the quality of life of patients.^2^ A growing body of evidence shows that tinnitus is not a cochlear lesion but a neurological symptom closely related to abnormal brain remodeling.^3-5^ For example, studies have shown that tinnitus can lead to significant structural and functional alterations in the brain that may cause or at least contribute to the clinical symptoms of tinnitus.^3,5-7^ To date, many hypotheses have been advanced for the mechanisms of brain remodelling that may contribute to tinnitus, such as the Bayesian inference, upregulated spontaneous neuronal firing, dysfunctional noise cancelling system, tonotopic map reorganization, increased central noise, increased neural synchrony and aberrant neural connectivity within or outside the auditory pathway.^8-12^ However, none of these hypotheses can fully explain the mechanism underlying tinnitus, and unsurprisingly, no adequate treatment has yet been developed for tinnitus.

The insula, an important region in the limbic system, has been implicated to play a vital role in tinnitus, especially for its secondary functional abnormalities, such as tinnitus-related distress and anxiety.^13^ Neuroimaging investigations, including functional MRI (fMRI) and EEG, have also proved this view.^14-19^ For instance, increased responses in the anterior insula may indicate successful adaptation to tinnitus perception, and greater synchrony of EEG signals in the insula has been associated with more severe distress due to tinnitus.^15-18^ Increased regional brain activity and increased functional connectivity (FC) in the insula have been observed on fMRI in tinnitus patients.^14,19^ Grey matter (GM) atrophy and compromised circuit function have also been detected within the insula in tinnitus patients.^20,21^ Rauschecker *et al*.^21^ even found that the insula acts as a hub in the shared neural circuit of chronic pain and tinnitus. These studies showed that the insula, a core region of the limbic system for perceptual sensations, assigns affective significance to sensory stimuli and modulates the flow of information within the brain.^21^ In addition to being an important component of the limbic system, the insula is a core region of the salience network, which continuously monitors the external world and carefully determines the response of other brain networks to new information and stimuli. The insula can be divided into six subregions that correspond to distinct brain functions.^22,23^ The specific contributions of the different subregions of the insula to tinnitus and its secondary dysfunction and the underlying mechanisms are yet to be determined.

Although the process of tinnitus development is a dynamic one, few studies have investigated neural plasticity during the transition of recent-onset tinnitus to chronic tinnitus. The latest research on this topic was published by Lan *et al*.^24^; however, their study was an EEG-based whole-brain functional investigation and did not apply fMRI methods or focus on core brain areas such as the insula.

We hypothesized that significant differences exist in the reorganization of the insula structure and function between patients with recent-onset tinnitus and those with chronic tinnitus. Therefore, the current study aimed to clarify the role of the insula and its subregions during the transition of recent-onset tinnitus to chronic tinnitus and reveal the possible underlying mechanism. For this purpose, we analysed the alterations in structure, regional brain activity and FC among patients with recent-onset tinnitus, those with chronic tinnitus and healthy control (HC) subjects by using voxel-based morphometry, fractional amplitude of low-frequency fluctuation (fALFF) measurements^25^ and seed-based FC analysis. We then studied the relationships between the neuroimaging indices and the patients’ clinical features.

## Materials and methods

### Ethics statement and study subjects

Ethical approval for the present study was obtained from the ethics committee of Beijing Friendship Hospital, Capital Medical University (approval no. 2017-P2-134-01). The study was conducted in accordance with the tenets of the Declaration of Helsinki, and each subject provided written informed consent before participating in the study.

We enrolled patients with recent-onset or chronic idiopathic tinnitus and HCs in this study. Consistent with previous studies, we took 1 year (not included) as the cut-off between recent-onset and chronic tinnitus.^26-28^ Except for the duration of tinnitus, the inclusion and exclusion criteria were identical to those used in our previous work (no patients with hyperacusis).^3,5,29^ In particular, most patients did not have hearing loss, as confirmed using pure tone audiometry (air conduction hearing thresholds at 125–8000 Hz, with octave intervals). The severity of tinnitus and its functional consequences were quantified (from 0 to 100 points) using the Tinnitus Handicap Inventory (THI). Participants completed the THI before undergoing neuroimaging. Self-rating Anxiety Scale (SAS) and Self-rating Depression Scale (SDS) were used to evaluate patients’ anxiety and depression. All HCs underwent pure tone audiometry to rule out hearing impairment as well. All exclusion criteria were applied to the HCs, and no abnormalities were found.

### MRI protocols

Both the tinnitus patients and the HC subjects underwent structural and functional brain imaging on a 3.0 T Prisma MRI scanner (Siemens, Erlangen, Germany); a head coil (64 channels, phased array) was used during scanning. All scanning sequences, parameters and the processing flow for the subjects during the scanning process have been described in detail in one of our previous studies.^3^

### Definition of insula subregions

**Figure 1:**
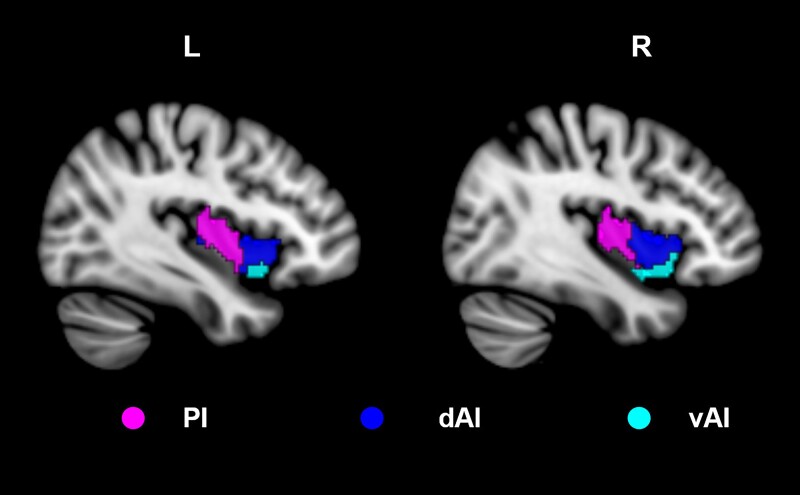
**Insula subregions.** The insula was divided into three subregions in each hemisphere: the PI, the dAI and the vAI.

According to the results of a connection-based parcellation study,^23^ the insula was divided into a total of six subregions, three per hemisphere: posterior insula (PI), dorsal anterior insula (dAI) and ventral anterior insula (vAI; Fig. 1). The insula template was obtained from the corresponding author by email.

### Processing of structural data

All high-resolution structural data were checked, and scans with any artefacts (e.g. susceptibility artefacts and artefacts due to head movement or equipment malfunction) were excluded. The remaining images were subjected to the following pre-processing steps by applying the CAT12 package (http://www.neuro.uni-jena.de) based on SPM 12:

- Segmentation. The images were segmented into white matter (WM), GM and CSF regions.
- Normalization. Using diffeomorphic anatomical registration and the CAT12 default algorithm, we individually normalized the WM and GM components to the standard Montreal Neurological Institute space and then multiplied the normalized components by the non-linear part of the deformation field to measure the relative GM and WM volumes.
- Smoothening. An 8 mm full width at half maximum Gaussian kernel was used to smoothen the GM/WM volume images.
- Extraction. The mean GM volumes of the insula and each insula subregion were extracted and compared among the three study groups by using one-way analysis of covariance (ANCOVA; SPSS version 23.0, IBM, Armonk, NY, USA), with age and sex as covariates. Differences between pairs of groups were evaluated using *post hoc* analysis (*P* < 0.05).

### Processing of functional images and FC analysis

Processing of the resting-state fMRI scans was accomplished using Data Processing & Analysis for (Resting-State) Brain Imaging (http://www.rfmri.org/dpabi).^30^ The pre-processing procedure has been described in detail in our previous study.^22^ In brief, pre-processing involved the following steps: removal of the first 10 volumes of the time series, slice timing, realignment (maximum translation or rotation less than 2.5 mm or 2.5°), normalization to the Montreal Neurological Institute template and resampling to isotropic 3 mm × 3 mm × 3 mm, smoothening with a 6 mm full width at half maximum Gaussian kernel, regression of 26 nuisance covariates (WM, CSF signals and Friston 24 head motion) and band-pass filtering (0.01–0.08 Hz). Next, FC analysis was performed with the insula and its subregions as seeds. The time series of voxels within each region were averaged to create a reference time series for each seed. We performed correlation analyses within the six insula subregions as well as between the seven seeds and the remaining voxels in the whole brain. To improve normality, we applied the Fisher *r*-to-*z* transformation. Differences in FC among the three study groups were analysed using the general linear model in SPM12 with total intracranial volume, age and sex serving as covariates [voxel-level uncorrected *P* < 0.0001; cluster-level *P* < 0.05 with family-wise error (FWE) correction]. FC differences in pairs of study groups were evaluated using *post hoc* analysis (*P* < 0.05, FWE correction).

### fALFF calculation

The process of fALFF followed the procedure employed in our previous work.^31^ After pre-processing of the functional data (excluding the step of band-pass filtering), fALFF calculations were performed using Data Processing & Analysis for (Resting-State) Brain Imaging pipeline.^30^ The main steps were as follows: (i) To acquire the power spectrum, we transformed the time series to a frequency domain by using fast Fourier transforms (FFTs; with 0% taper and minimum length). (ii) To determine the ALFF, we calculated the square root of the power spectrum averaged across the frequencies of 0.01–0.08 Hz in each voxel. (iii) To eliminate individual differences, the calculated ALFF values (square roots above) were standardized by dividing each value by the global mean ALFF. (iv) The fALFF was defined as the ratio of the power spectrum of the ALFF range (0.01–0.08 Hz) to the power spectrum of the entire frequency range (0–0.25 Hz).^25^ Then, we extracted the fALFF value of the insula and its six subregions for the subsequent group comparison by using the insula subregions template as masks.

### Statistical analysis

We verified the homogeneity and normality of variance of the data before analysing the extracted values. One-way ANCOVA and *post hoc* analysis were used for normally distributed data, while Kruskal–Wallis *H* test was used for non-normally distributed data, with age and sex as covariates. Potential associations of the structural and functional alterations in the insula and its subregions with the clinical characteristics of tinnitus patients were explored using partial correlation analysis after adjustments for age and sex.

## Results

### Clinical characteristics

We enrolled a total of 92 subjects in this study: 24 patients with recent-onset idiopathic tinnitus (<12 months), 32 patients with chronic idiopathic tinnitus (>12 months) and 36 HCs. Of these, two subjects (one patient and one HC) were excluded due to excessive head motion (>2.5 mm and 2.5°) in the functional data pre-processing. Thus, the functional analysis only included 55 patients with idiopathic tinnitus and 35 HCs. The clinicodemographic characteristics of the tinnitus patients and HC subjects are shown in Table 1. No significant between-group differences were found in age, gender, handedness and tinnitus laterality and pitch (*P* > 0.05, uncorrected). In particular, we found no significant difference in the THI score, which was the main factor for evaluating tinnitus severity, between the recent-onset and chronic tinnitus patients. Furthermore, no differences were observed in the SAS and SDS scores, which were used to evaluate anxiety and depression.

**Table 1:** Demographic and clinical characteristics of the tinnitus patients and HCs

Demographic	Recent-onset (*n* = 24)	Chronic (*n* = 32)	HCs (*n* = 36)	*P*-value
Age, years	45.63 ± 12.32	48.06 ± 9.96	46.61 ± 10.01	0.688^a^
Gender (M/F)	16/8	21/11	24/12	0.995^b^
Handedness (right/left)	24/0	32/0	36/0	>0.99^a^
THI score	60.75 ± 29.51	53.75 ± 22.97	NA	0.341^c^
SAS	42.58 ± 8.79	44.31 ± 11.94	NA	0.552^c^
SDS	48.83 ± 10.12	47.72 ± 10.99	NA	0.699^c^
Duration (month)	≤12	≥36	NA	NA
Tinnitus pitch	250–8000 Hz	250–8000 Hz	NA	NA
Laterality (right/left/bilateral)	9/5/10	6/3/23	NA	0.075^b^

F, female; M, male; NA, not applicable; SAS, Self-rating Anxiety Scale; SDS, Self-rating Depression Scale; THI, Tinnitus Handicap Inventory. ^a^One-way ANOVA. ^b^Chi-square test. ^c^Two-sample *t*-test.

### Morphological changes in the insula and its subregions

**Figure 2:**
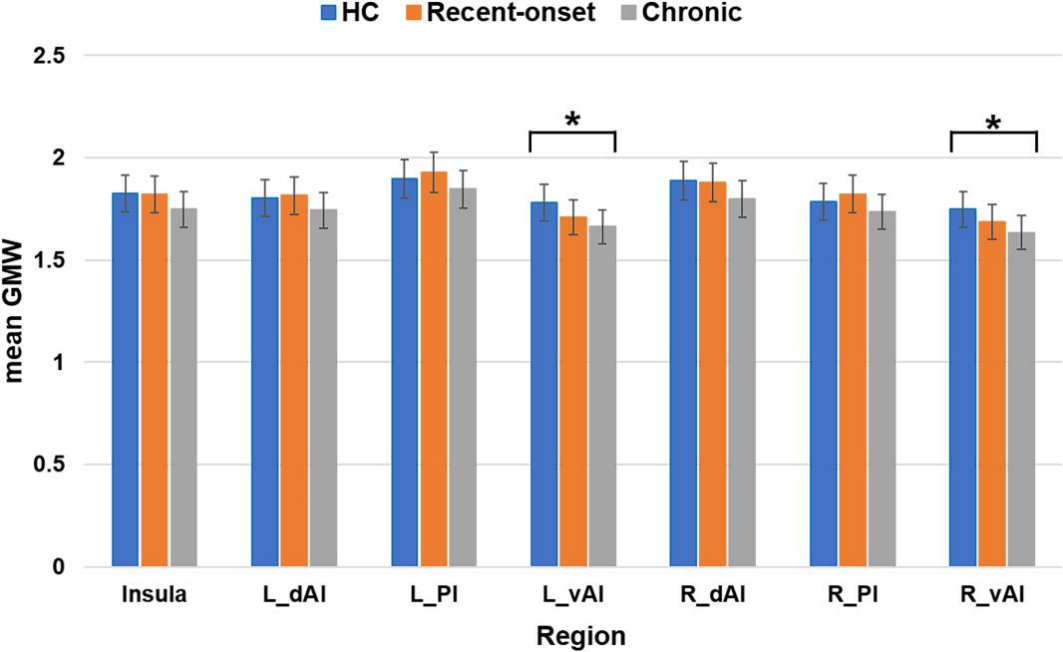
**Morphological changes in the insula and its subregions.** Comparisons among the three groups showed significant differences in the GM volume of only the vAI. *Post hoc* analysis showed bilateral GM atrophy in the vAI in patients with chronic tinnitus as compared to HCs (*P* < 0.05, uncorrected). No significant differences were observed in the other insula subregions and in the whole insula among the three groups. *Indicates significant differences (*P* < 0.05, uncorrected).

The *post hoc* analysis revealed a bilateral decrease in the GM volume of vAI in patients with chronic tinnitus as compared with the HCs (*P* < 0.05, uncorrected). No significant differences in GM volume were found for the other insula subregions or for the insula itself among the three study groups (*P* > 0.05, uncorrected) (Table 2; Fig. 2).

**Table 2:** Group differences in GM volume of the insula and its subregions among the recent-onset and chronic tinnitus patients and HCs

One-way ANCOVA					
Brain region	Recent-onset (*n* = 24) (M ± SD)	Chronic (*n* = 32) (M ± SD)	HCs (*n* = 36) (M ± SD)	*F*-value	*P*-value (uncorrected)
Insula	1.82 ± 0.19	1.75 ± 0.16	1.83 ± 0.24	1.462	0.237
L-dAI	1.81 ± 0.19	1.74 ± 0.16	1.80 ± 0.23	1.083	0.343
L-PI	1.93 ± 0.21	1.85 ± 0.21	1.90 ± 0.26	0.904	0.409
L-vAI	1.71 ± 0.19	1.66 ± 0.16	1.78 ± 0.24	3.888	0.024*
R-dAI	1.88 ± 0.20	1.80 ± 0.19	1.89 ± 0.26	1.554	0.217
R-PI	1.82 ± 0.21	1.74 ± 0.19	1.78 ± 0.25	1.085	0.342
R-vAI	1.69 ± 0.18	1.63 ± 0.16	1.75 ± 0.23	3.854	0.025*

*Post hoc*				
Brain region	HCs—recent-onset (MD ± SE)	*P* value (uncorrected)	HCs—chronic (MD ± SE)	*P*-value (uncorrected)
L-vAI	0.08 ± 0.04	0.055	0.10 ± 0.04	0.010*
R-vAI	0.07 ± 0.04	0.070	0.10 ± 0.04	0.009*

dAI, dorsal anterior insula; HCs, healthy controls; L, left; M, mean; MD, mean difference; PI, posterior insula; R, right; SD, standard deviation; SE, standard error; vAI, ventral anterior insula. * represents a statistically significant difference (*P* < 0.05, uncorrected).

### Alterations in the regional brain activity of the insula and its subregions

**Figure 3:**
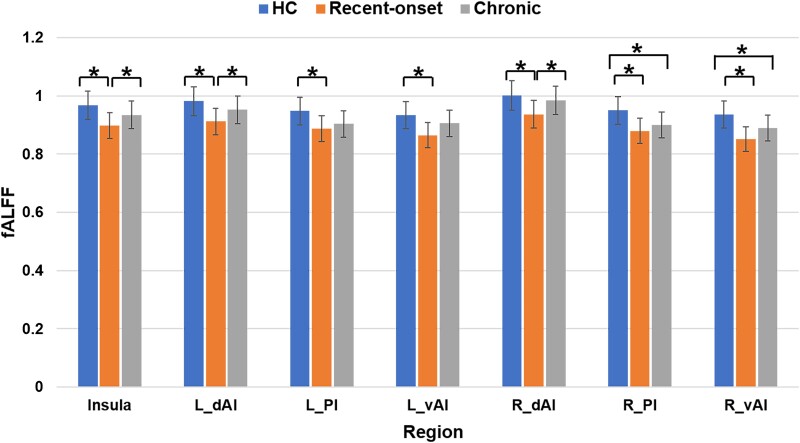
**Alterations in the regional brain activity of the insula and its subregions.** One-way ANCOVA and *post hoc* analysis showed that the fALFF was decreased in the insula, right and left dAI, right PI, and right vAI in patients with recent-onset and chronic tinnitus relative to HCs. Only recent-onset tinnitus patients demonstrated decreased fALFF in the left and right dAI as compared with the HCs. *Indicates significant differences (*P* < 0.05, uncorrected).

One-way ANCOVA and *post hoc* analysis revealed that, compared with the HCs, both patient groups showed decreased fALFF in the right PI and right vAI, while only the recent-onset tinnitus group showed decreased fALFF in the left PI and left vAI. Moreover, the recent-onset patient group showed decreased fALFF in the insula and bilateral dAI compared with HCs and decreased fALFF in the same regions relative to the chronic group (*P* < 0.05, uncorrected) (Table 3; Fig. 3).

**Table 3:** Group differences in fALFF of the insula and its subregions among the recent-onset and chronic tinnitus patients and HCs

One-way ANCOVA					
Brain region	Recent-onset (*n* = 24) (M ± SD)	Chronic (*n* = 32) (M ± SD)	HCs (*n* = 36) (M ± SD)	*F*-value	*P*-value (uncorrected)
Insula	0.90 ± 0.09	0.94 ± 0.06	0.97 ± 0.06	7.143	0.001*
L-dAI	0.91 ± 0.10	0.95 ± 0.06	0.98 ± 0.07	6.422	0.003*
L-PI	0.89 ± 0.10	0.90 ± 0.97	0.95 ± 0.81	3.510	0.034*
L-vAI	0.86 ± 0.12	0.91 ± 0.07	0.93 ± 0.08	4.644	0.012*
R-dAI	0.94 ± 0.09	0.98 ± 0.06	1.00 ± 0.07	7.280	0.001*
R-PI	0.88 ± 0.11	0.90 ± 0.11	0.95 ± 0.09	3.989	0.022*
R-vAI	0.85 ± 0.12	0.89 ± 0.08	0.94 ± 0.08	6.803	0.002*

*Post hoc*		* *		* *		* *
Brain region	HCs—recent-onset (MD ± SE)	*P* value (uncorrected)	HCs—chronic (MD ± SE)	*P* value (uncorrected)	Recent-onset –Chronic (MD ± SE)	*P* value (uncorrected)
Insula	0.07 ± 0.02	0.000*	0.03 ± 0.02	0.08	−0.04 ± 0.02	0.036*
L-dAI	0.07 ± 0.02	0.001*	0.03 ± 0.02	0.151	−0.05 ± 0.02	0.029*
L-PI	0.06 ± 0.02	0.016*	0.04 ± 0.02	0.054	−0.02 ± 0.02	0.503
L-vAI	0.07 ± 0.02	0.003*	0.03 ± 0.02	0.199	−0.04 ± 0.02	0.070
R-dAI	0.07 ± 0.02	0.000*	0.01 ± 0.02	0.409	−0.05 ± 0.02	0.005*
R-PI	0.07 ± 0.03	0.009*	0.05 ± 0.02	0.050*	−0.02 ± 0.03	0.399
R-vAI	0.09 ± 0.02	0.000*	0.04 ± 0.02	0.044*	−0.04 ± 0.02	0.081

dAI, dorsal anterior insula; HCs, healthy controls; L, left; M, mean; MD, mean difference; PI, posterior insula;

R, right; SD, standard deviation; SE, standard error. vAI, ventral anterior insula. * represents a statistically significant difference (*P* < 0.05, uncorrected).

### Alterations in FC of the insula and its subregions

We found significant differences in FC among the three groups by using the insula and its subregions as seeds. Compared with the HCs, the recent-onset and chronic tinnitus patients showed significant decreases in FC in the following regions when the insula or its subregions were used as seeds: (i) insula (seed), inferior frontal gyrus (IFG), cingulate gyrus (CG) and hippocampus; (ii) left dAI (seed), IFG and CG; (iii) left PI (seed), middle temporal gyrus (MTG) and CG; (iv) left vAI, insula, anterior CG, superior frontal gyrus (SFG), middle frontal gyrus, IFG, inferior parietal lobule and orbitofrontal cortex (OFC); (v) right dAI (seed), IFG, superior temporal gyrus (STG), CG, hippocampus and insula; (vi) right PI, STG and CG; and (vii) right vAI, IFG and anterior CG (*P* < 0.05, FWE correction) (Supplementary Table 1; Supplementary Fig. 1). In addition, when the right dAI was used as a seed, increased FC was found in the insula in recent-onset and chronic tinnitus patients as compared with the HCs (*P* < 0.05, FWE correction).

**Figure 4:**
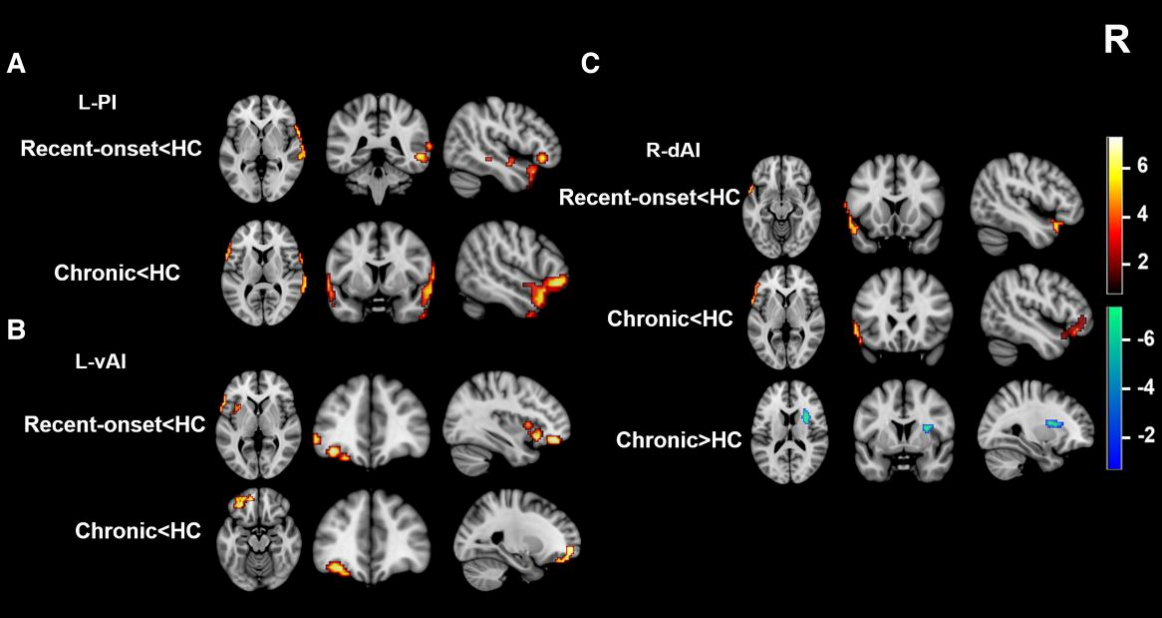
**Group differences of alterations in the FC of the insula and its subregions between the recent-onset and chronic patient groups.** (**A**) Compared with the HCs, the recent-onset tinnitus patients showed decreased FC in the right MTG, while the chronic tinnitus patients showed decreased FC in the left IFG and right STG when the left PI (L-PI) was used as a seed. (**B**) Recent-onset tinnitus patients showed decreased FC in the left insula, left IFG and left OFC, while chronic tinnitus patients showed decreased FC in the left SFG when the left vAI (L-vAI) was used as a seed. (**C**) Recent-onset tinnitus patients demonstrated decreased FC in the left STG, while chronic tinnitus patients showed decreased FC in the left IFG and increased FC in the right insula when the right dAI (R-dAI) was used as a seed (*P* < 0.05, FWE correction).

In addition, compared with the HCs, only recent-onset tinnitus patients showed decreased FC in the following brain regions: (**A**) left PI (seed), right MTG; (**B**) left vAI (seed), left insula, left IFG and left OFC; and (**C**) right dAI (seed) and left STG (*P* < 0.05, FWE correction) (Table 4; Fig. 4). Furthermore, only chronic tinnitus patients showed decreased FC in the following regions: (**A**) left PI (seed), left IFG and right STG; (**B**) left vAI (seed) and left SFG; and (**C**) right dAI (seed) and left IFG (*P* < 0.05, FWE correction) (Table 4; Fig. 4). Analysis with the right dAI as a seed also showed increased FC in the right insula (**C**) of chronic tinnitus patients as compared with the HCs (*P* < 0.05, FWE correction) (Table 4; Fig. 4). We did not find any statistical differences in FC within the insula (among the six subregions) in the three groups (*P* > 0.05, uncorrected).

**Table 4:** Brain regions of abnormal FC with insula and its subregions between the recent-onset and chronic tinnitus patients

Brain region	Cluster size (voxels)	Peak *T*-score	MNI coordinates (mm)		
			*x*	*y*	*z*
**L-PI**					
**Recent-onset < HC**					
R middle temporal gyrus	508	5.61	57	15	−12
**Chronic < HC**					
L inferior frontal gyrus	244	6.96	−60	12	3
R superior temporal gyrus	492	6.10	60	12	−9
**L-vAI**					
**Recent-onset < HC**					
L insula	58	5.83	−36	18	−9
L inferior frontal gyrus	66	4.82	−54	18	0
L OFCant	58	4.49	−36	39	−15
**Chronic < HC**					
L superior frontal gyrus	107	4.59	−24	54	−3
**R-dAI**					
**Recent-onset < HC**					
L superior temporal gyrus	139	5.83	−57	12	−6
**Chronic < HC**					
L inferior frontal gyrus	150	5.80	−60	12	3
**Chronic > HC**					
R insula	43	4.81	30	3	15

The threshold was set at a *P* < 0.05 (FWE corrected). dAI, dorsal anterior insula; FWE, family wise error; L, left; OFCant, anterior orbitofrontal cortex; PI, posterior insula; R, right; vAI, ventral anterior insula.

### Correlations between brain imaging measurements and clinical features

**Figure 5:**
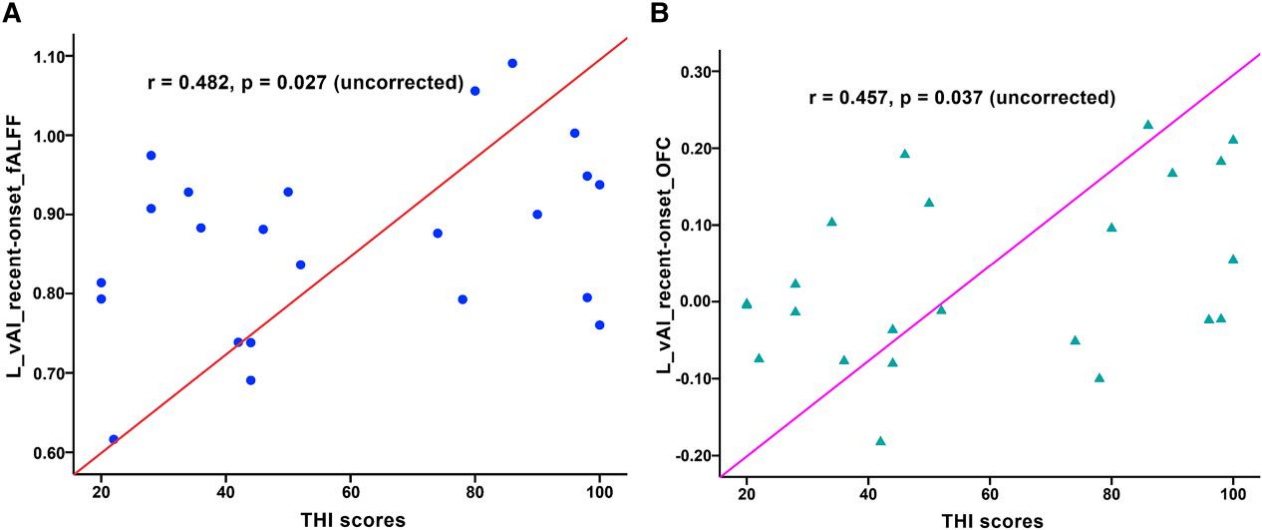
**Correlations between brain imaging measurements and clinical features.** Partial correlation analysis indicated that both the fALFF value of the left vAI (L-vAI; *r* = 0.482, *P* = 0.027, uncorrected; (**A**) and the strength of the FC between the L-vAI and the left OFC (*r* = 0.457, *P* = 0.037, uncorrected; (**B**) were positively correlated with the THI scores in recent-onset tinnitus patients.

In the recent-onset tinnitus group, partial correlation analysis indicated that THI scores were positively correlated with the left vAI fALFF value (*r* = 0.482, *P* = 0.027, uncorrected; Fig. 5A) and the strength of the FC between the left vAI and the left OFC (*r* = 0.457, *P* = 0.037, uncorrected; Fig. 5B). No other significant correlations were found between clinical factors and brain imaging properties in the recent-onset or chronic tinnitus patients (*P* > 0.05, uncorrected).

## Discussion

The present study focused on the role of the insula and its subregions in the transition from recent-onset to chronic tinnitus from the structural and functional perspectives. A major finding of this study is that compared with HCs, both recent-onset and chronic tinnitus patients showed decreased regional activities in the insula and most of its subregions as well as abnormal FC of the seeds (the insula and its subregions) with the limbic system, auditory-related regions and frontal regions. In particular, bilateral GM-volume decrease in the vAI and fALFF reduction in the left PI and left vAI were solely observed in chronic and recent-onset tinnitus patients, respectively. When using the left PI, left vAI and right dAI as seeds for whole-brain FC analysis, we found that recent-onset tinnitus patients showed decreased FC in the right MTG, left IFG, left OFC, left STG and left insula, while chronic tinnitus patients demonstrated decreased FC in the left IFG, left SFG, left IFG and right STG and increased FC in the right insula.

The differences in the structural and functional alterations in the insula between patients with recent-onset and chronic idiopathic tinnitus are important for identifying the central nervous changes in these two conditions and may provide novel insights into the mechanism of tinnitus generation, especially for the progression of recent-onset tinnitus to chronic tinnitus.

### Morphological and regional activity alterations in the insula and its subregions

In terms of structural changes, we only found a bilateral decrease in the GM volume of the vAI in chronic tinnitus patients as compared with the HCs. We found no structural differences between recent-onset tinnitus patients and the HCs in any of the insula subregions. The vAI is connected to the limbic system, participates in affective processes^32^ and even contains a socio-emotional region.^33^ The anterior insula is considered to be critically involved in subjective feelings and emotional experiences,^34^ such as anxiety in traumatic brain injury patients.^35^ Thus, the bilateral vAI GM atrophy in chronic tinnitus patients may be a consequence of their long-term suffering from the negative emotions (such as anxiety and depression) caused by tinnitus,^36^ unlike recent-onset tinnitus patients.^37^

In terms of the regional activity alterations, both recent-onset and chronic tinnitus patients exhibited abnormal fALFF across the whole insula, though the changes were more severe in the recent-onset group. As a crucial part of the central gatekeeping system for perceptual sensations, the insula helps to assign affective values to sensory stimuli and to modulate the flow of information in the brain.^21^ Compromised circuit function of the insula has been reported in tinnitus^20,21^; as patients attend to their phantom auditory sensation, a corresponding decrease occurs in the activation of the insula. Thus, while the consequences of recent-onset tinnitus, such as anxiety and depression, may be severe in some patients, chronic patients may develop a certain degree of adaptation. This may explain why the recent-onset tinnitus group showed greater changes in regional brain activity (in the left PI and left vAI). We also found that the THI score was positively correlated with the fALFF value of the left vAI in recent-onset tinnitus patients. In other words, the more obvious the changes in fALFF in the left vAI, the more serious the clinical symptoms of patients. This finding further indicates that the symptoms of recent-onset tinnitus patients might be more severe than those of chronic tinnitus patients, though further study is needed to confirm this.

### FC alterations in the insula and its subregions

In this study, abnormal connectivities between the insula subregions and auditory/non-auditory-related brain areas were observed in both recent-onset and chronic tinnitus patients. Nonetheless, significant differences were found between the recent-onset and chronic tinnitus groups, mainly in certain areas of the frontal cortex, auditory-related cortex and the core hub of the limbic system. The frontal cortex, specifically the SFG, IFG and OFC, may contribute to some perceptual features of tinnitus.^38^ As a main integrative hub of the tinnitus brain circuit, the SFG receives and integrates all types of information from various brain regions in response to internal and external stimuli.^38^ As the core region of response inhibition, the IFG might mirror the attempt to control the bottom-up attention allocation to the tinnitus percept in a top-down way.^39^ The OFC is considered to be closely linked to depression and even cognitive and emotional impairment.^40^ The STG and MTG, as important parts of the auditory centre, are related to many other complex functions, such as language, multisensory integration and semantic memory.^41^ Moreover, abnormal neuronal activity or FC of the MTG may be associated with disruption of the default mode network in patients with tinnitus.^38,42^ The limbic system, including hub regions such as the amygdala, insula and anterior cingulate cortex, responds to emotional stimulation and is implicated in memory.^43^ Numerous studies have indicated increased connectivity of limbic and auditory regions in tinnitus.^13,44^ Thus, tinnitus may be caused by damage to the limbic system making limbic–auditory interactions inefficient and leading to a failure to inhibit these interactions.^45,46^ We also found a positive correlation of the mean THI score with the strength of the FC between the left vAI and left OFC in the recent-onset tinnitus group, which may indicate that the clinical symptoms of recent-onset tinnitus patients are more severe than those of chronic tinnitus patients, as indicated by the fALFF data. However, during FC analysis, we did not find significant differences between the six insula subregions in the three study groups. A possible reason for this is that tinnitus may be more likely to induce abnormal connectivity of the insula and its subregions with the rest of the brain than to change the fixed internal networks of the insula, though the underlying mechanism is unclear.

Taken together, the above abnormal connectivities demonstrate that the progression from recent-onset tinnitus to chronic tinnitus is a dynamic process. The FC of the insula subregions with the auditory-related cortex, frontal cortex and limbic system, as a common tinnitus-related circuit, is crucially involved in tinnitus occurrence and development.

Some limitations of this study should be noted. First, this study had a cross-sectional design and a relatively small sample size, that’s why the brain structural and regional brain activity changes were not statistically corrected, but the results demonstrate at least a trend of insula remodelling in tinnitus; hence, the conclusions must be verified in a larger population. Second, more left-handed subjects should be enrolled in future studies to rule out the influence of handedness on the study findings. Third, many of the patients had different degrees of hearing loss (although it may be the cause of their tinnitus), and the tinnitus lateralization varied among the patients. In our future study, we will try to exclude the impacts of hearing loss and tinnitus lateralization on the results. Certainly, it would be quite interesting to explore the effects of tinnitus lateralization and hearing loss on brain reorganization in recent-onset and chronic tinnitus patients. Fourth, although all normal subjects reported no hearing loss or hyperacusis, we should still apply the same means as tinnitus patients to measure their hearing condition in future research.

## Conclusions

In conclusion, as the tinnitus duration increased (from less than 1 year to more than 1 year), we found that structural alterations and changes in neural activity in the insula subregions, and the connectivity of the insula subregions with areas in the frontal cortex, limbic system and temporal cortex changed continuously. Our findings indicate that tinnitus generation and development occur in a dynamic manner, and the structure, regional activities and related connectivity changes of the insula and its subregions play a crucial role in this process. In particular, abnormal connectivity of the insula subregions with areas in the auditory cortex, frontal cortex and limbic system, as a tinnitus-related circuit, may predict the progression from recent-onset tinnitus to chronic tinnitus. The above regions may serve a vital role in the central mechanism of tinnitus.

## Supplementary material

Supplementary material is available at *Brain Communications* online.

## Supplementary Material

fcad261_Supplementary_Data

## Data Availability

The data sets generated for this study are available on request to the corresponding authors.
