# Supplementary material for: Role of insula and its subregions in progression from recent onset to chronic idiopathic tinnitus
Source: Brain Commun. 2023 Oct 7;5(5):fcad261. doi: 10.1093/braincomms/fcad261 (PMC10586310; doi:10.1093/braincomms/fcad261)
Supplement: fcad261_Supplementary_Data [file fcad261_supplementary_data.docx]

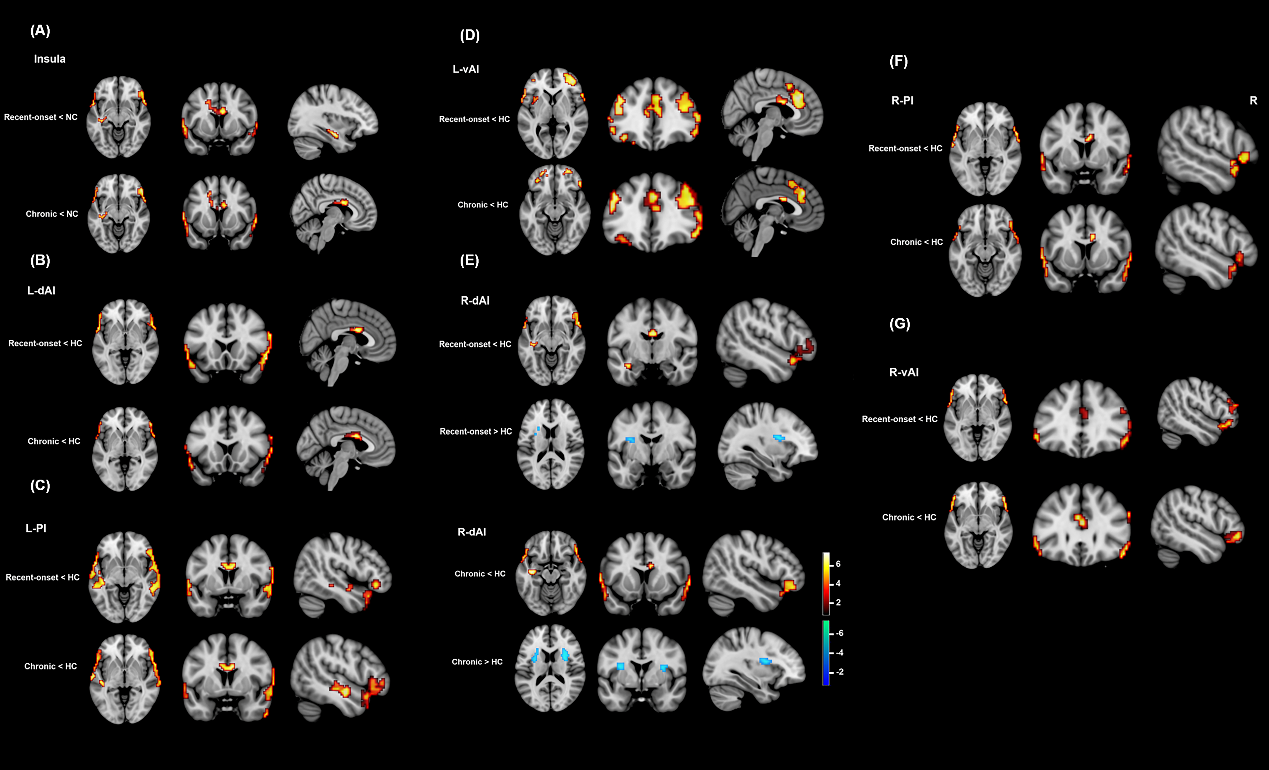


**Supplementary figure**

**Supplementary figure1.** **Alterations in the functional connectivity (FC) of the insula and its subregions among the recent-onset patient group, chronic patient group, and HCs**

**(A)** Compared with the healthy controls (HCs), both the recent-onset and chronic tinnitus patients showed decreased FC in the bilateral inferior frontal gyrus (IFG), cingulate gyrus, and left hippocampus when the insula was used as a seed. **(B)** Compared with the HCs, both the recent-onset and chronic tinnitus patients showed decreased FC in the bilateral IFG and cingulate gyrus when the left dorsal anterior insula (L-dAI) was used as a seed. **(C)** Compared with the HCs, recent-onset tinnitus patients showed decreased FC in the right middle temporal gyrus (MTG), chronic tinnitus patients showed decreased FC in the left IFG and right superior temporal gyrus (STG), both the two groups showed decreased FC in the left MTG and cingulate gyrus when the left posterior insula (L-PI) was used as a seed. **(D)** Compared with the HCs, recent-onset tinnitus patients showed decreased FC in the left insula, left IFG, and left anterior orbitofrontal cortex, chronic tinnitus patients showed decreased FC in the left superior frontal gyrus (SFG), both the two groups showed decreased FC in the anterior cingulate, bilateral middle frontal gyrus (MFG), right inferior parietal lobule (IPL) when the left ventral anterior insula (L-vAI) was used as a seed. **(E)** Compared with the HCs, recent-onset tinnitus patients showed decreased FC in the left STG, chronic tinnitus patients showed decreased FC in the left IFG and increased FC in the right insula, both the two groups showed decreased FC in the right IFG, cingulate gyrus, left hippocampus and increased FC in the left insula when the right dorsal anterior insula (R-dAI) was used as a seed. **(F)** Compared with the HCs, both the recent-onset and chronic tinnitus patients showed decreased FC in the bilateral STG and cingulate gyrus when the right posterior insula (R-PI) was used as a seed. **(G)** Compared with the HCs, both the recent-onset and chronic tinnitus patients showed decreased FC in the bilateral IFG and anterior cingulate when the right ventral anterior insula (R-vAI) was used as a seed.

**Supplementary table1.** **Brain regions of abnormal functional connectivity with insula and its subregions among the recent-onset, chronic tinnitus patients and HCs.**

| **Brain region** | **Cluster size**  **(voxels)** | **Peak T-score** | **MNI Coordinates (mm)** | | |
| --- | --- | --- | --- | --- | --- |
|  |  |  | **x** | **y** | **z** |
| **Insula** |  |  |  |  |  |
| **Recent-onset** **< HC** |  |  |  |  |  |
| R Inferior frontal gyrus | 187 | 6.08 | 57 | 18 | -9 |
| L Inferior frontal gyrus | 153 | 5.90 | -57 | 12 | -6 |
| Cingulate gyrus | 159 | 5.29 | -3 | 3 | 24 |
| L Hippocampus (aal3v1) | 41 | 4.79 | -33 | -15 | -15 |
| **Chronic < HC** |  |  |  |  |  |
| R Inferior frontal gyrus | 197 | 5.73 | 54 | 33 | -6 |
| L Inferior frontal gyrus | 144 | 5.63 | -60 | 12 | 9 |
| L Hippocampus (aal3v1) | 49 | 4.96 | -36 | -12 | -18 |
| Cingulate gyrus | 123 | 4.95 | -3 | 0 | 24 |
| **L-dAI** |  |  |  |  |  |
| **Recent-onset < HC** |  |  |  |  |  |
| L Inferior frontal gyrus | 134 | 6.32 | -57 | 12 | -6 |
| R Inferior frontal gyrus | 198 | 6.11 | 57 | 18 | -9 |
| Cingulate gyrus | 132 | 5.52 | -3 | 0 | 24 |
| **Chronic < HC** |  |  |  |  |  |
| Cingulate gyrus | 127 | 5.35 | -3 | 0 | 24 |
| L Inferior frontal gyrus | 73 | 5.22 | -60 | 12 | 9 |
| R Inferior frontal gyrus | 97 | 4.96 | 57 | 18 | -9 |
| Frontal_Inf_Tri_R (aal3v1) | 25 | 4.63 | 63 | 18 | 24 |
| **L-PI** |  |  |  |  |  |
| **Recent-onset < HC** |  |  |  |  |  |
| L Middle temporal gyrus | 464 | 5.68 | -66 | -9 | 15 |
| **R Middle temporal gyrus** | **508** | **5.61** | **57** | **15** | **-12** |
|  |  |  |  |  |  |
| Cingulate gyrus | 101 | 5.32 | 6 | 6 | 27 |
| **Chronic < HC** |  |  |  |  |  |
| **L Inferior frontal gyrus** | **244** | **6.96** | **-60** | **12** | **3** |
| **R Superior temporal gyrus** | **492** | **6.10** | **60** | **12** | **-9** |
| L Middle temporal gyrus | 177 | 4.91 | -48 | -12 | -15 |
| Cingulate gyrus | 131 | 4.77 | -15 | -12 | 36 |
| **L-vAI** |  |  |  |  |  |
| **Recent-onset** **< HC** |  |  |  |  |  |
| **L insula** | **58** | **5.83** | **-36** | **18** | **-9** |
| L Anterior cingulate | 559 | 5.56 | 6 | 30 | 30 |
| R Middle frontal gyrus | 688 | 5.24 | 33 | 39 | 15 |
| **L Inferior frontal gyrus** | **66** | **4.82** | **-54** | **18** | **0** |
| R Inferior parietal lobule | 163 | 4.82 | 54 | -54 | 51 |
| **L OFCant (aal3v1)** | **58** | **4.49** | **-36** | **39** | **-15** |
| L Middle frontal gyrus | 159 | 4.41 | -36 | 48 | 15 |
| **Chronic < HC** |  |  |  |  |  |
| R Middle frontal gyrus | 594 | 5.28 | 39 | 54 | 3 |
| R Anterior cingulate | 363 | 5.11 | 3 | 33 | 33 |
| **L Superior frontal gyrus** | **107** | **4.59** | **-24** | **54** | **-3** |
| L Middle frontal gyrus | 135 | 4.20 | -45 | 36 | 21 |
| R Inferior parietal lobule | 61 | 3.89 | 57 | -57 | 48 |
| **R-dAI** |  |  |  |  |  |
| **Recent-onset < HC** |  |  |  |  |  |
| R Inferior frontal gyrus | 181 | 6.04 | 57 | 18 | -9 |
| **L Superior temporal gyrus** | **139** | **5.83** | **-57** | **12** | **-6** |
| Cingulate gyrus | 105 | 5.13 | -3 | -3 | 24 |
| L Hippocampus | 39 | 4.72 | -36 | -12 | -18 |
| **Chronic < HC** |  |  |  |  |  |
| **L Inferior frontal gyrus** | **150** | **5.80** | **-60** | **12** | **3** |
| R Inferior frontal gyrus | 203 | 5.79 | 54 | 33 | -6 |
| L Hippocampus | 50 | 4.57 | -36 | -15 | -15 |
| Cingulate gyrus | 98 | 4.50 | -3 | 0 | 24 |
| **Recent-onset > HC** |  |  |  |  |  |
| L insula | 33 | 4.46 | -30 | 3 | 18 |
| **Chronic > HC** |  |  |  |  |  |
| L insula | 46 | 5.82 | -30 | 0 | 18 |
| **R insula** | **43** | **4.81** | **30** | **3** | **15** |
| **R-PI** |  |  |  |  |  |
| **Recent-onset < HC** |  |  |  |  |  |
| L Superior temporal gyrus | 95 | 5.14 | -60 | 9 | 3 |
| R Superior temporal gyrus | 147 | 5.12 | 60 | 15 | -3 |
| Cingulate gyrus | 31 | 4.26 | 9 | 9 | 30 |
| **Chronic < HC** |  |  |  |  |  |
| L Superior temporal gyrus | 100 | 5.69 | -60 | 9 | 0 |
| R Superior temporal gyrus | 151 | 6.30 | 60 | 12 | -9 |
| Cingulate gyrus | 48 | 4.56 | -6 | -12 | 24 |
| **R-vAI** |  |  |  |  |  |
| **Recent-onset < HC** |  |  |  |  |  |
| R Inferior frontal gyrus | 174 | 5.68 | 57 | 18 | -6 |
| L Inferior frontal gyrus | 76 | 5.26 | -57 | 12 | -6 |
| Anterior cingulate | 49 | 4.54 | 6 | 24 | 24 |
| **Chronic < HC** |  |  |  |  |  |
| R Inferior frontal gyrus | 164 | 5.01 | 51 | 36 | -9 |
| L Inferior frontal gyrus | 79 | 4.94 | -60 | 12 | 3 |
| Anterior cingulate | 22 | 3.80 | -6 | 30 | 24 |

Note: The threshold was set at a p < 0.05 (FWE corrected). HCs, healthy controls; L, left; R, right; PI, posterior insula; dAI, dorsal anterior insula; vAI, ventral anterior insula; OFCant, anterior orbitofrontal cortex, FWE, family-wise error.
